# Evaluation of the prevalence of the most common psychiatric disorders in patients with type 2 diabetes mellitus using the patient health questionnaire: results of the cross-sectional “DIA2PSI” study

**DOI:** 10.1007/s00592-022-01993-x

**Published:** 2022-11-07

**Authors:** Angelo Emilio Claro, Clelia Palanza, Marianna Mazza, Andrea Corsello, Alessandro Rizzi, Linda Tartaglione, Chiara de Waure, Giuseppe Marano, Simone Piciollo, Giovanna Elsa Ute Muti Schuenemann, Marta Rigoni, Paola Muti, Alfredo Pontecorvi, Luigi Janiri, Gabriele Sani, Dario Pitocco

**Affiliations:** 1 Fondazione Policlinico Universitario A. Gemelli IRCCS, Largo Agostino Gemelli, 8-CAP 00168 Rome, Italy; 2 Department of Psychiatry, Università Cattolica del Sacro Cuore, Largo Agostino Gemelli, 8-CAP 00168 Rome, Italy; 3 Istituto Italiano Di Antropologia-ISItA, Piazzale Aldo Moro, 5-00185 Rome, Italy; 4 Diabetes Care Unit, Department of Endocrinology and Diabetes, Università Cattolica del Sacro Cuore, Fondazione Policlinico Universitario Agostino Gemelli IRCCS, Largo Agostino Gemelli, 8-CAP 00168 Rome, Italy; 5 Department of Medicine and Surgery, University of Perugia, Piazza dell’ Università, 1, 06123 Perugia, Italy; 6 Associazione Italiana di Psicologia Giuridica–AIPG, Via Bisagno, 15, 00199 Rome, Italy; 7 Health Research Methods, Evidence and Impact Department, McMaster University, McMaster University Medical Centre, 1280 Main Street West 2C Area, Hamilton, ON L8S 4K1 Canada; 8 Department of Biomedical, Surgical and Dental Sciences, University of Milan, Via Festa del Perdono 7, 20122 Milan, Italy; 9 Division of Endocrinology, Department of Endocrine-Metabolic and Dermo-Rheumatology, Fondazione Policlinico Universitario A. Gemelli IRCCS, Università Cattolica del Sacro Cuore, Largo Agostino Gemelli, 8-CAP 00168 Rome, Italy

**Keywords:** Diabetes Mellitus Type 2, Italy, Mental Disorders, Depression, Feeding and Eating Disorders, Alcohol-Induced Disorders

## Abstract

**Aims:**

Common Psychiatric Disorders (CPDs) are associated with the development of overweight and obesity, the strongest risk factors for the onset and maintenance of Type 2 Diabetes mellitus (T2D). To the best of our knowledge, this is the first study to assess the prevalence of CPDs in patients with T2D in Italy.

**Methods:**

This is a monocentric cross-sectional study; *n* = 184 T2D patients were screened for CPDs using the Patient Health Questionnaire (PHQ). Primary outcome was to evaluate the prevalence of CPDs. To assess association between CPDs and risk factors, we have utilized univariable logistic regression models.

**Results:**

64.1% were men, median age was 67 (59–64) and median BMI 27 (25–30) kg/m^2^. The 42.9% tested positive for one or more mental disorders, 25.6% for depression. Patients with higher BMI (*p* = 0.04) had an increased likelihood of testing positive to the PHQ. Patients who had implemented lifestyle changes (*p* < 0.01) and were aware that mental health is linked to body health (*p* = 0.07) had a reduction in the likelihood of testing positive.

**Conclusions:**

Prevalence of CPDs in T2D patients is higher than in the general population. Since CPDs favor the onset and subsistence of T2D, integrated diabetic-psychiatric therapy is required for improvement or remission of T2D in patients with comorbid CPDs.

**Supplementary Information:**

The online version contains supplementary material available at 10.1007/s00592-022-01993-x.

## Introduction

Across the world, both Diabetes and Psychiatric disorders are on the rise [1, 2]. A global Diabetes epidemic is ongoing, with 537 million of adult people currently affected, which is expected to reach 783 million by 2045 [1]. Diabetes strongly affects disability, mortality, and global health expenditure. It absorbs the 12% of global health expense [3] and has caused about 6.7 million deaths in 2021 [1]; Covid-19 in two years has caused 5.7 million of deaths [4]. Diabetes is one of the most important risk factors for cardiovascular disease, increasing cardiovascular risk in two- to fourfold [5], is the leading cause of blindness [6], chronic renal failure and dialysis [7], and of non-traumatic amputation of the lower limbs [8].

Type 2 Diabetes mellitus (T2D) determines the 95% of all cases of diabetes in the world [9]. In Italy, over 3 million 200 thousand people are affected by diabetes, 5.3% of the entire population [10].

About psychiatric disorders worldwide, it is estimated that 17.6% of population experienced a CPDs [11], and 4.4% suffer from depressive disorder [12].

Depression is the leading cause of disability worldwide [12], and over 700.000 people die due to suicide every year being the fourth leading cause of death in people aged 15–29 [13]. In primary care, in patients with physical illnesses, depression is one of the most frequent causes of consultation [14].

In Italy, 5.4% of the population suffer from a depressive disorder [15]and 7.3% experience a CPDs [16].

Regarding the connection between CPDs and T2D, it is recognized that CPDs are associated with the development of overweight and obesity[17], the strongest risk factors for the development of T2D [18–20].As the worldwide prevalence of both diabetes and mental health problems is increasing rapidly and is expected to do it further in the upcoming years [1] and given the link between these two classes of diseases, it is urgent to have studies evaluating the epidemiological situation of CPDs in patients with T2D.

The aim of this study was to assess the prevalence of the CPDs in patients with T2D, in a sample of patients numerically representative of the Italian population. Furthermore, we hypothesized that it would have been possible to identify factors that favor the positivity for a psychiatric disorder in the analyzed population. To the best of our knowledge, this is the first study to assess the prevalence of the CPDs in patients with T2D, in a sample of patients numerically representative of the Italian population.

### Subjects, materials and methods

Monocentric cross-sectional study, conducted at the Fondazione Policlinico Universitario Agostino Gemelli IRCCS, UCSC, Rome, Italy (FPUAG).

A full description of the protocol used in the present study is provided in the Online Resource 1.

In brief, between October 2020 and November 2021 a sample of consecutive outpatient clinic attendees with T2D at the Diabetes Care Unit of the FPUAG was invited to participate in the study, with the aim of including 184 people with T2D.

### Inclusion/exclusion criteria

Inclusion criteria were age between 18 and 85 years and diagnosis of T2D [21]. Exclusion criteria were diagnosis of Type 1 Diabetes mellitus (T1D); inability to complete the survey tools because of cognitive difficulties; to be very sick and unable to read and understand Italian. Women who were pregnant or had given birth in the last 6 months were also excluded.

Prior to interview, the site investigators fulfilled an information form for each patient. This form included socio-demographic information such as age, gender, civil status, residence location, educational level, and smoke status. T2D-specific information and anthropometric profile have been registered: duration of T2D, height, weight and BMI at diagnosis of T2D; actual height weight and BMI; antidiabetic drugs or insulin prescription; family history of T2D and history of T2D complications (cardiovascular disease, retinopathy, peripheral neuropathy, and renal disease); hypertension. Measurements of blood pressure, HbA1c, were also recorded. A section on past psychiatric history and on awareness of the importance of lifestyle changes and of mental health with respect to weight control and the prevention of physical diseases is also contained within the form; for more details, see Online resource 2. The patient's medical history was obtained before the PHQ administration to adequately attribute the presence of any physical symptoms to a medical condition or to the somatoform disorder. Any prescribed medicine for mental health problem was noted, as was any diagnosis or therapy of any psychiatric disorder. Patients were queried if they lived in a rural or an urban area and declared their highest level of education. Marital status was defined as married/co-habiting vs being single/widowed/divorced.

Each participant was requested to complete the Patient Health Questionnaire. The Italian full version of the PHQ [22] was used for psychiatric screening, as it covers the CPDs. The PHQ is a self-administered questionnaire that allows to evaluate the presence of specific disorders: major depressive disorder, other depressive disorders, panic attack disorder, other anxiety disorders, bulimia nervosa, binge eating disorder, dependence or alcohol abuse.

In this study, all the sections, from 1 to 11, of the official Italian version of the PHQ were used.

Subjects diagnosed with any psychiatric disorders were directed to consult their physician or the Psychiatric Unit of FPUAG for further evaluation and treatment.

### Ethical approval

The study protocol was approved by the Ethics Committee (EC) of the FPUAG Università Cattolica del Sacro Cuore, on 08/08/2020 with number ID 3298 and protocol number 0040703/20. All procedures performed in the study were in accordance with the ethical standards of the institutional committee and with the Declaration of Helsinki and its later amendments. All participants signed a written informed consent form.

### Statistical analysis

Sample size calculation was performed assuming an expected prevalence of psychiatric disorders of 37.5%, setting up an accuracy of 7% and a confidence level of 97%, thus calculating 184 patients to administer the PHQ. The expected prevalence was estimated from a previous study carried out in T2D patients [23].

Included patients were described in clinical and demographic characteristics through descriptive statistics techniques. Continuous variables had been checked for normality with Shapiro–Wilk test. Therefore, normally distributed data were expressed as mean and standard deviation (SD), and non-normally with median and first and third quartiles (q1–q3). Dichotomous variables, categorical variables, and scores were expressed as numbers and percentages.

Primary outcome was the prevalence of common psychiatric disorders in patients with T2D assessed through the Patient Health Questionnaire.

To evaluate potential risk/protective factors associated with patients’ psychiatric disorders positivity, we performed univariable logistic regression models. For each factor, we calculated the Odds Ratio (OR) with 95% Confidence Interval (CI) of being positive to PHQ. Statistical analyses were performed with Stata software, and statistical significance cut-off was set *p* < 0.05.

### Data availability

The data associated with the study are available from the corresponding author on reasonable request.

## Results

**Fig. 1:**
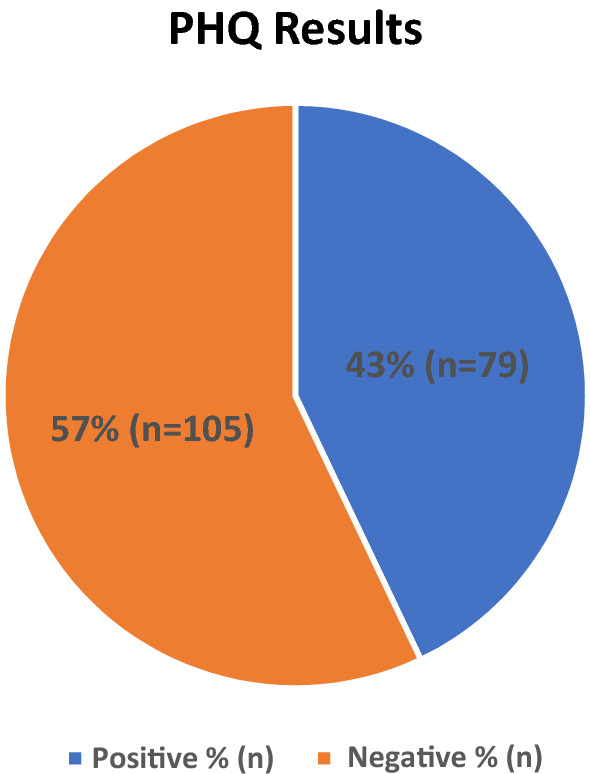
Prevalence of CPDs in patients with T2D from PHQ

The results are based on the analysis of 240 patients enrolled in the study (Fig. 1-Online resource 3).

A total of 240 individuals recruited with T2D, agreed to participate in the study, with a response rate of 80%. The main reason for not participating was being too busy to undergo the examination; furthermore, 31 patients were excluded due to uncertain T2D diagnosis, whereas other 25 were excluded because they left the PHQ incomplete. Thus, 184 patients were analyzed.

### Baseline characteristics

The demographic characteristics of the patients interviewed are reported in Table 1. Patients were 118 men (64.1%) and 66 women (35.9%), the median age was 67 years (q1–q3: 59–64), and 139 were married/co-habiting (75.5%). About the residence location, 116 (63.1%) were living in Urban location, while 68 (36.9%) in rural area. It was also recorded that patients had an average 11-year study education (± 4 years of study) and 162 (88.0%) were no current smoker.

**Table 1:** Demographic Profile

Demographic profile	
Total patients’ number	184
Male, n (%)	118 (64.1)
Female, n (%)	66 (35.9)
Age, median (q1–q3), years	67 (59–64)
*Marital status*	
Married/co-habiting, n (%)	139 (75.5)
Widow/Widower, n (%)	19 (10.4)
Single, n (%)	14 (7.6)
Divorced, n (%)	12 (6.5)
*Residence Location*	
Urban location, n (%)	116 (63.1)
Rural location, n (%)	68 (36.9)
*Educational level*	
Elementary school, n (%)	18 (9.9)
Middle school, n (%)	54 (29.3)
High school, n (%)	84 (45.6)
Higher education, n (%)	28 (15.2)
*Smoke status*	
Cigarette, n (%)	20 (10.9)
E-cig, n (%)	2 (1.1)
No Current smoker, n (%)	162 (88.0)

*E-cig* Electronic cigarettes

The population evaluated have (Table 2): T2D on average from 10 years (q1–q3: 5–17). The median weight recorded was 80 kg (69–87) with a median height of 170 cm (162–176), median BMI 27 kg/m2 (25–30), of which 53 patients (28.8%) had a BMI in the obesity range. The weight and BMI of the patients at the time of T2D diagnosis were also collected and were: median weight 80 kg (q1–q3: 70–92) median BMI: 29 kg/m2 (25–31), of which 73 patients (39.7%) had a BMI in the obesity range. Additional values collected at the time of test administration were: median HbA1c and pharmacological therapy. Patients had median HbA1c of 6.8 (q1–q3: 6.1–7.6), 51 mmol/mol, and 146 patients (79.4%) were taking some type of oral and injectable (non-insulin) antidiabetic agents for the treatment of T2D, while 64 (34.8%) patients were taking insulin. A total number of 118 (64.1%) persons have at the time of the interview at least one complication related to T2D: 81 (44%) patients have CVD, 32 (17.4%) had neuropathy, 57 (40%) patients have retinopathy, 27 (14.7%) patients have Chronic Kidney Disease (CKD), and 140 (76.1%) suffer from hypertension.

**Table 2:** T2D specific and anthropometric profile

Diabetes mellitus specific and anthropometric profile	
Diabetes mellitus duration, median (q1–q3), years	10 (5–17)
Current	
Height, median (q1–q3), cm	170 (162–176)
Weight, median (q1–q3), kg	80 (69–87)
BMI, median (q1–q3), kg/m^2^	27 (25–30)
BMI ≥ 30 kg/m^2^, n (%)	53 (28.8)
At Diagnosis	
Weight, median (q1–q3), kg	80 (70–92)
BMI, median (q1–q3), kg/m^2^	29 (25–31)
BMI ≥ 30 kg/m^2^, n (%)	73 (39.7)
Drugs	
Oral and Injectable (Non-Insulin) Pharmacological Agents for the Treatment of T2D, n (%)	146 (79.4)
Insulin, n (%)	64 (34.8)
T2D related complications	
Patients with at least 1 T2D related complication, n (%)	118 (64.1)
Retinopathy, n (%)	57 (40.0)
Neuropathy, n (%)	32 (17.4)
CKD, n (%)	27 (14.7)
Cardiovascular, n (%)	81 (44)
Number of T2D related complications	
0, n (%)	66 (35.9)
1, n (%)	61 (33.2)
2, n (%)	38 (20.6)
3, n (%)	16 (8.7)
4, n (%)	3 (1.6)
Hypertension	
Hypertension, n (%)	140 (76.1)
HbA1C	
HbA1C, median (q1–q3)%	6.8 (6.1–7.6)
HbA1C ≥ 6.5%, n (%)	120 (65.2)

q1 = first quartile; q3 = third quartile

### Patient health questionnaire results

**Fig. 2:**
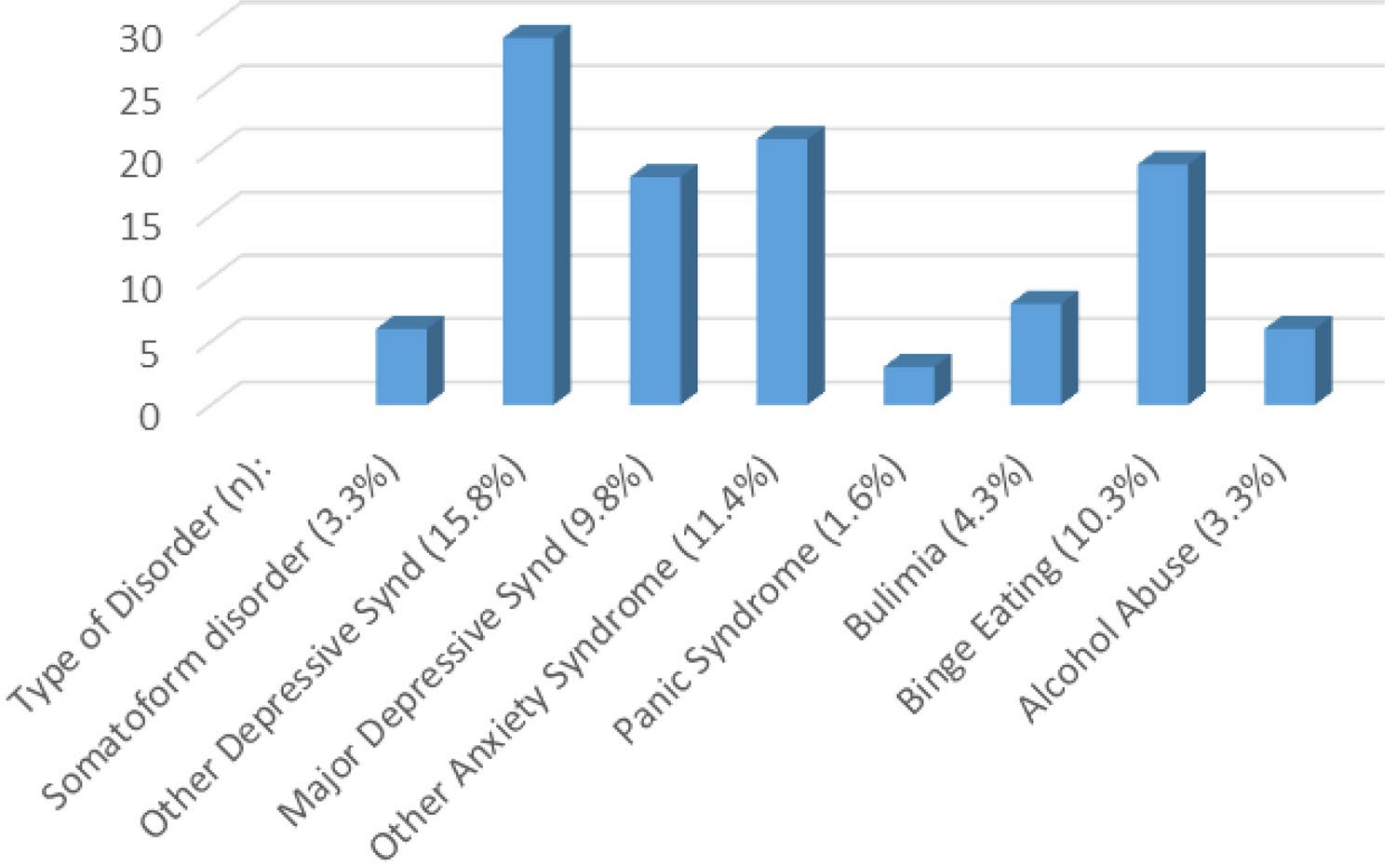
Type of disorder diagnosed with PHQ

The prevalence of the different psychiatric disorders evaluated through the PHQ is provided in Fig. 1–2 and Table 3.

**Table 3:** Psychological results of DIA2PSI participants according to the patient health questionnaire (PHQ)

Psychological profile	
Total patients number	184
PHQ	
Positive, n (%)	79 (42.9)
Negative, n (%)	105 (57.1)
Type of Disorder	
Somatoform disorder, n (%)	6 (3.3%)
Other Depressive Syndrome, n (%)	29 (15.8%)
Major Depressive Syndrome, n (%)	18 (9.8%)
Other Anxiety Syndrome, n (%)	21 (11.4)
Panic Syndrome, n (%)	3 (1.6%)
Bulimia, n (%)	8 (4.3%)
Binge Eating, n (%)	19 (10.3%)
Alcohol Abuse, n (%)	6 (3.3%)
Quality of life	
Quality of life 1, n (%)	144 (78.3)
Quality of life 2, n (%)	27 (14.7)
Quality of life 3, n (%)	8 (4.3)
Quality of life 4, n (%)	5 (2.7)
Aware that mental health linked to body health?	
Yes, (%)	134 (72.8)
No, n (%)	50 (27.2)
Previous PSY treatment?	
Yes, n (%)	46 (25.0)
No, n (%)	138 (75.0)
Previous Pharmaco-PSY treatment?	
Yes, n (%)	38 (20.6)
No, n (%)	146 (79.4)
Aware that change lifestyle improves diabetes mellitus?	
Yes, n (%)	174 (94.6)
Has implemented lifestyle changes, n (%)	145 (78.8)

Of the sample examined, 79 people out of 184 tested positives for the PHQ, namely 42.9%, and 25.6% tested positive for a depressive disorder (percentage derived from the sum of Other Depressive Syndrome and Major Depressive Syndrome). The last question of the PHQ is: "If you checked off any problems on this questionnaire, how difficult have these problems made it for you to do your work, take care of things at home, or get along with other people?" to this question alternatives answer are: not difficult at all, somewhat difficult, very difficult, extremely difficult. 144 (78.3%) patients answered: not difficult at all.

In addition to the collection of socio-demographic data, and to the administration of the PHQ, patients were asked about their awareness of the importance of weight loss in the remission of T2D [24, 25] and if they “are aware about the importance of mental health in controlling body weight and in prevention of physical diseases?”.

On the importance of lifestyle changes in the T2D remission, 174 people (94.6% of patients) replied that they received this information from their treating physician, while the percentage dropped to 134 patients (72.8%) when they were asked if, besides the importance of physical health, they know the importance of mental health in body weight control and prevention of physical diseases.

The patients were also asked whether they presented a psychiatric diagnosis in medical history, or if they had ever been treated by a psychiatrist or a psychotherapist and/or if they ever took psychopharmacological treatments in the past. The 46 patients (25.0%) had been treated by a psychiatrist or psychotherapist, and among them, 38 patients (20.6%) ​​had undertaken psychopharmacological treatments.

### Factors associated with positivity to the PHQ

Demographic and lifestyle predictors of current common psychiatric disorder are shown in Table 4 in Online resource 3.

Among all demographic and lifestyle variables we have analyzed, our results showed that female in comparison with males had 72% higher probability of testing positive to PHQ (OR = 1.72; 95%CI: 0.94–3.17), even if the result was not significant (*p* = 0.08). For every increase of 1 year in patients’ age, we noticed a 2% of reduction in developing positivity to the questionnaire (OR = 0.98; 95%CI: 0.95–1.00), but the result is not statistically significant (*p* = 0.07). BMI is a risk factor associated with a greater likelihood of testing positive for PHQ, indeed for every 1-point increase in current BMI there is a 7% increased risk (OR = 1.07; 95%CI: 1.00–1.14, *p* = 0.04), and for every 1-point increase in BMI at diagnosis the OR is equal to 1.05 (95%CI: 1.00–1.10, *p* = 0.06). Urban residence location, in comparison with rural, showed a 42% probability reduction in association with CPDs (OR = 0.58; 95%CI: 0.31–1.06), but it was not statistically significant (*p* = 0.07). Civil status and educational level did not show significant results.

Diabetes specific and psychosocial predictors of current common psychiatric disorder are shown in Table 5–6 in Online resource 3. Among those, antidiabetic drugs (non-insulin) use was associated with a 30% reduction in probability of having some common psychiatric disorder (OR = 0.70; 95%CI:0.34–1.43, *p* = 0.32), but the result was not statistically significant. Patients taking insulin, in comparison with patients that did not, had an 89% increase in the likelihood of testing positive to the PHQ (OR = 1.89; 95%CI: 1.02–3.49, *p* = 0.04), and this finding is likely related to the severity of the diabetic disease. Other interesting results are that patients who had implemented lifestyle changes showed a 62% odds reduction in being diagnosed of psychiatric disorders (OR = 0.38; 95%CI: 0.18–0.79, *p* < 0.01), patients who are aware that mental health is linked to body health had a 46% odds reduction (OR = 0.54; 95%CI: 0.28–1.04, *p* = 0.07), and patients who had Previous or actual Pharmaco-PSY treatment had a 246% probability increase in testing positive to the PHQ (OR = 2.46; 95%CI: 1.19–5.12, *p* = 0.02).

## Discussion

We have observed substantial rates of mental health problems, that is, the 43% of patients treated at the FPUAG Diabetes Care Unit tested positive for a CPDs. The most common disorders detected were depressive syndrome, in almost 26% of patients, anxiety syndromes (11.4%) and binge eating (10.3%). Other psychiatric disorders less frequently present were: bulimia (4.3%), alcohol abuse (3.3%), somatoform disorder (3.3%), panic syndrome (1.6%).

The risk factors probably associated with the positivity to the test were: female sex, higher BMI, insulin intake, previous psychopharmacological treatment. Protective factors were aging, lifestyle changes implementation, awareness that mental health is related to body health.

Patients taking insulin and patients with higher BMI showed a statistically significant increase in the odds of testing positive for PHQ. These findings could suggest that people with psychiatric disorders are less able to adhere to prescriptions for T2D therapy and remission [26].

Other interesting results were that patients who had implemented lifestyle changes (*p* < 0.01) and patients who were aware that mental health is linked to body health were less likely to test positive to the PHQ, while patients who had previous or actual psychopharmacological treatment had a 246% probability increase in testing positive to the PHQ. These data seem to confirm that patients who were not affected by a mental disorder and were aware that mental health is linked to body health showed better adherence to lifestyle changes and therapies necessary for T2D treatment and remission.

Furthermore, the proportion of the DIA2PSI sample who had been or was still being treated by a mental health professional was 25%. At least 18% of the sample was neither diagnosed nor treated for a psychiatric disorder. These data confirm that people with T2D are underdiagnosed and undertreated for psychiatric disorders [26].

It is important to assess the prevalence of CPDs in T2D patients as it is recognized that a wide range of psychiatric disorders have a higher prevalence in subjects with T2D than in the general population [27]. Furthermore, most of the studies so far have focused their attention on the coexistence of T2D and depression, not evaluating other CPDs [28]. DIA2PSI is one of the few studies to address this topic. DIA2PSI results on depression are in line with other studies in the literature which found that depressive disorders are more common in people with T2D than in the general population [29]. Indeed, we have observed substantial higher rate of CPDs and depression in T2D patients (42.9% and 29%) then in the general Italian population (17.6% and 4.4%). Instead, making a comparison between the results of our study and other studies that have evaluated the presence of eating disorders, anxiety disorders, somatoform disorder and alcohol abuse in patients with T2D is difficult because studies on this type of disorders are rare in the literature [17, 27, 28]. In particular, lack of studies evaluating eating disorders appears quite surprising given that overeating is one of the fundamental factors underlying the onset and maintenance of T2D [18, 19].

It is necessary to assess psychiatric comorbidity in patients with T2D since CPDs are able to induce overweight, obesity the strongest risk factors for T2D [17–19]. Clinical trials have shown that weight loss ≥ 15% is necessary in the first years of T2D diagnosis to achieve disease remission [24, 25]. Nevertheless, the incidence of T2D remission in routine care settings is infrequent (< 1%) [30], in particular in patients with depressive symptoms [31]. Indeed, depression favors weight gain and determines a 60% increased risk of developing T2D [32–34] directly through three of its symptoms that we have re-named Bridge Symptoms (BS): alteration of eating behavior [35, 36], reduced physical activity [37, 38] and inadequate sleep duration [39, 40].

Also in CPDs, Bridge Symptoms are present increasing the risk of developing T2D [17]. In both depression and CPDs, there are other pathological mechanisms that can lead to increased risk of developing T2D: systemic chronic inflammation, increased insulin resistance, decreased insulin release from pancreatic beta cells and cognitive mechanisms like decreased ability to think and to focus, anhedonia, weariness, and lack of motivation that negatively affect the probability of attending medical examination and check-up or sustaining a physically active lifestyle [17].

Furthermore, people with T2D are responsible for much of the management of their disease,but if they have depressive symptoms, these lead to poor self-managementand early development of negative outcomes including early mortality [26, 41].

## Conclusions

Performing psychiatric screening in patients with T2D is needed to intercept cases of mental disorder that might otherwise remain unknown and to intervene on the development of T2D and its remission, considering how CPDs favor its onset and maintenance [17, 30, 42]. This could help reduce the T2D epidemic, driving a reduction in Years of Life Lost (YLL) given that Diabetes is the 9th cause of death worldwide, and it is an important contributing cause to the first and second cause of death globally (ischemic heart disease and stroke).

Moreover, this can lead to substantial economic savings given that T2D absorbs 12% of global health expenditure [3].

### Limitations

The population analyzed was mainly Caucasian, which limits generalization to non-Caucasian populations such as South Asians, who tend to develop T2D with less weight gain. Quality and robustness of our findings are limited by cross-sectional study design, restricted sample size, monocentric study, and the lack of multivariable analysis. Also, as with any diagnostic test, the PHQ does not detect all cases of mental disorders.

## Supplementary Information

Below is the link to the electronic supplementary material.

Supplementary file1 (DOCX 69 kb)

Supplementary file2 (DOCX 16 kb)

Supplementary file3 (DOCX 35 kb)

## Data Availability

The data associated with the study are available from the corresponding author on reasonable request.
