# Supplementary material for: Evaluation of the prevalence of the most common psychiatric disorders in patients with type 2 diabetes mellitus using the patient health questionnaire: results of the cross-sectional “DIA2PSI” study
Source: Acta Diabetol. 2022 Nov 7;60(2):247–55. doi: 10.1007/s00592-022-01993-x (PMC9640892; doi:10.1007/s00592-022-01993-x)
Supplement: Supplementary file 1 — Supplementary file1 (DOCX 69 kb) [file 592_2022_1993_MOESM1_ESM.docx]

**Title**

Evaluation of the prevalence of the most common psychiatric disorders in patients with type 2 diabetes mellitus using the Patient Health Questionnaire: results of the cross-sectional “DIA2PSI” study.

**Journal Name**

Acta Diabetologica

**Author names**

Angelo Emilio Claro ^1,2^*, Clelia Palanza ^3^, Marianna Mazza ^1,2^, Andrea Corsello ^4^, Alessandro Rizzi ^4^, Linda Tartaglione ^4^, Chiara de Waure ^5^, Giuseppe Marano ^1,2^, Simone Piciollo ^6^, Giovanna E. U. Muti Schuenemann ^7^, Marta Rigoni ^8^, Paola Muti ^8^, Alfredo Pontecorvi ^9^, Luigi Janiri ^1,2^, Gabriele Sani ^1,2^, Dario Pitocco ^4^.

*Corresponding Author: email – dott.claro@gmail.com

^1^ Fondazione Policlinico Universitario A. Gemelli IRCCS, Largo Agostino Gemelli, 8 - CAP 00168, Rome, Italy.

^2^ Department of Psychiatry, Università Cattolica del Sacro Cuore, Largo Agostino Gemelli, 8 - CAP 00168, Rome, Italy.

**Protocol**

**Date 08/08/2020 Version n. 3**

**Evaluation of the prevalence of the most common psychiatric disorders in patients with type 2 diabetes using the Patient Health Questionnaire: results of the cross-sectional “DIA2PSI” study.**

**Abstract**

**Background**: A wide range of psychiatric disorders are more prevalent in individuals with Type 2 Diabetes Mellitus (T2D) than in the general population. Most of the studies, so far, have focused on the coexistence of diabetes and depression, based on self-administered questionnaires developed specifically for psychiatric patients.

**Aim of the study:** To evaluate the prevalence of psychiatric disorders, in a sample of consecutive outpatient clinic attendees with T2D at the Diabetes Care Unit of the Fondazione Policlinico Universitario Agostino Gemelli IRCCS, UCSC, Rome, Italy (FPUAG) using a scale developed specifically for the screening of psychiatric diseases in internal medicine patients the PHQ.

**Inclusion Criteria:** Age between 18 and 85 aa, Diagnosis of T2D.

**Statistical Plan**: the achievement of the primary objective, assuming an expected prevalence of 37.5%, with an accuracy of 7% and a confidence level of 97% is guaranteed by a sample size of n = 184 patients.

**Data analysis:** the sample will be described through descriptive statistical techniques. The qualitative variables will be represented by tables of absolute and percentage frequencies. The quantity will be summarized through measurements of minimum, maximum, range, mean and standard deviation. Continuous variables will be checked for normality with Shapiro Wilk test.

**Date:** 08 August 2020

**Version:** 3

This study protocol has been designed and will be conducted in accordance with the principles enshrined in the Good Clinical Practice (GCP) guidelines as applicable and in compliance with the Declaration of Helsinki and current guidelines for observational studies.

Summary

[Synopsis 4](#_Toc18742526)

[Introduction and rationale](#_Toc18742527) 5

……………………………………………………………………………………………………………………………………………………………………….

[Study objectives 6](#_Toc18742528)

[Study design, duration and setting 6](#_Toc18742529)

[Materials and methods 7](#_Toc18742530)

[Inclusion criteria 7](#_Toc18742531)

[Exclusion criteria 7](#_Toc18742532)

[Study procedures 7](#_Toc18742533)

[Statistical considerations 8](#_Toc18742534)

[Sample size 8](#_Toc18742535)

[Data Analysis 8](#_Toc18742536)

[References 8](#_Toc18742537)

# Synopsis

| **Title** | Evaluation of the prevalence of the most common psychiatric disorders in patients with type 2 diabetes using the Patient Health Questionnaire: results of the cross-sectional “DIA2PSI” study. |
| --- | --- |
| **Acronym** | DIA2PSI |
| ***Protocol number*** | 0040703/20 |
| ***EudraCTNumber*** | [N/A](https://it.wikipedia.org/wiki/N/A) |
| ***NCT number (clinicaltrials.gov)*** | N/A |
| **Version** | 3 |
| **Date** | 08/08/2020 |
| **Place of the study** | Fondazione Policlinico Universitario Agostino Gemelli IRCCS, UCSC, Rome, Italy (FPUAG) |
| **PI and CoPI** | PI: Prof. Luigi Janiri |
| **Study design** | Cross sectional study |
| **Blinding** | None |
| **Primary objective** | Evaluation of the prevalence of psychiatric disorders, in a sample of consecutive outpatient clinic attendees with T2D at the Diabetes mellitus Unit of the Fondazione Policlinico Universitario Agostino Gemelli IRCCS, UCSC, Rome, Italy (FPUAG) using a scale developed specifically for the screening of psychiatric diseases in general internal medicine patients, the PHQ. |
| **Secondary objective** | None |
| **Primary endpoint** | The primary objective will be achieved through the calculation of the prevalence of psychiatric disorders in patients with T2D. |
| **Secondary endpoint** | Organize a permanent psychiatric screening at the Diabetes Care Units of the FPUAG for patients with T2D, to initiate an integrated psychiatric-diabetic for people with T2D and who have a comorbid psychiatric disorder. |
| **Rational** | It is known that a wide range of psychiatric disorders have a higher prevalence in individuals with T2D than in the general population. However, so far, most studies have focused on the co-presence of T2D and depression based on self-administered questionnaires developed specifically for psychiatric patients. |
| **Inclusion criteria** | -Age between 18 and 85 aa  -Diagnosis of T2D according to the American Diabetes Association (ADA) |
| **Exclusion criteria** | -Diagnosis of Type 1 diabetes mellitus  -Inability to complete the survey tools because of cognitive difficulties  -To be very sick and unable to read and understand Italian  -Women who were pregnant or had given birth in the last 6 months were also excluded. |
| **Study procedures** | - fulfill an information form for each patient.  -Patients who agree to join the study will be requested to complete the Patient Health Questionnaire. |
| **Sample size** | the achievement of the primary objective, assuming an expected prevalence of 37.5%, with an accuracy of 7% and a confidence level of 97% is guaranteed by a sample size of n = 184 patients. The values taken into account are based on the few studies carried out and present in the literature which report a prevalence of psychiatric comorbidities of 37.5% in patients with T2D. |
| **Statistical analysis** | The patients included will be described in their clinical and demographic characteristics through descriptive statistics techniques. Continuous variables will be checked for normality with Shapiro Wilk test. Therefore, normally distributed data will be express as mean and standard deviation (SD), and non-normally with median and first and third quartiles (q1-q3). Dichotomous variables, categorical variables, and scores we will be express as numbers and percentages.  Primary outcome is the prevalence of common psychiatric disorders in patients with T2D assessed through the Patient Health Questionnaire. Moreover, to evaluate potential risk/protective factors associated with patients’ psychiatric disorders positivity, we will perform univariable logistic regression models. Therefore, for each factor, we will calculate the Odds Ratio (OR) with 95% Confidence Interval (CI) of being positive to PHQ. Statistical analyses will perform with Stata software, and statistical significance cut-off was set p<0.05. |

# Introduction and rationale

According to 2016 Istat data, there are over 3,2 million people with diabetes in Italy, 5.3% of the entire population, 16.5% among people over 65^1^ . The etiology of T2D is related to a diabetogenic lifestyle (excessive caloric intake, visceral and central obesity, and reduced physical activity). Although, according to the ADA guidelines, among the cornerstones of T2D therapy there is the increase of physical activity and healthy eating habits^2^, known and hypothetically feasible primary and secondary prevention interventions, it is estimated a continued increase in the obese/overweight population and people with T2D and in the years to come^3^. A wide range of psychiatric disorders have a higher prevalence in people with diabetes than in the general population^4,5^, but most studies present in the literature have evaluated the prevalence of psychiatric disorders in patients with both type 1 diabetes (T1D) and T2D, focusing mainly on the coexistence of depression and diabetes^6,7,8,9^ not considering other psychiatric disorders. In this study we will evaluate instead the prevalence of common psychiatric Disorders only in patients with T2D.

# Objectives of the study

The evaluation of the **prevalence** of common psychiatric disorders (not only depression) in a sample of consecutive outpatient clinic attendees with T2D at the Diabetes mellitus Unit of the Fondazione Policlinico Universitario Agostino Gemelli IRCCS, UCSC, Rome, Italy (FPUAG) using a scale developed specifically for the screening of psychiatric diseases in internal medicine patients the PHQ^10^; this tool, given the greater burden of somatic symptoms in internal medicine patients, is more suitable for identifying psychiatric disorders in this category of patients, than the scales developed for screening psychiatric disorders in psychiatric patients.

# Study design, duration and *setting*

Monocentric cross-sectional study. The study will take place at FPUAG, starting from October 2020. The recruitment and data collection period coincides. The study will last 1 year.

#

# Materials and methods

Patients aged between 18 and 85 years, diagnosed with T2D according to ADA^11^, belonging to the Diabetes Care Unit of the FPUAG will be recruited. The algorithm developed by the authors and supplied with the PHQ will be used for the coding of psychiatric diagnoses^10^. The PHQ is a self-administered questionnaire that allows to evaluate the presence of specific disorders: major depressive disorder, other depressive disorders, panic attack disorder, other anxiety disorders, bulimia nervosa, binge eating disorder, dependence, or alcohol abuse.

In this study all the sections, from 1-11, of the official Italian version of the PHQ will be used. The algorithm developed by the authors will be used to make the diagnosis with the PHQ^10^.

## Inclusion criteria

- Age between 18 and 85 aa

- Diagnosis of T2D according to the ADA

##

## Exclusion criteria

- Very sick patients unable to read in Italian were excluded

- Diagnosis of T1D

- Inability to complete the survey tools because of cognitive difficulties

- Be very sick and unable to read and understand Italian

- Women who were pregnant or had given birth in the last 6 months.

## Study procedures

Patients who agree to participate in the study will receive the PHQ test. Those who will test positive will be contacted for a psychiatric visit.

**Statistical considerations**

## Sample size

The achievement of the primary objective, i.e. the estimation of the prevalence of psychiatric diseases in patients suffering from T2D, assuming an expected prevalence of 37.5%, with an accuracy of 7% and a confidence level of 97% is guaranteed by a sample size equal to **N =** **184 patients** to which the PHQ test will be administered.

## Data analysis

The sample will be described in its clinical and demographic characteristics through descriptive statistical techniques. Continuous variables had been checked for normality with Shapiro Wilk test. Therefore, normally distributed data were expressed as mean and standard deviation (SD), and non-normally with median and first and third quartiles (q1-q3). Dichotomous variables, categorical variables, and scores were expressed as numbers and percentages.

Primary outcome was the prevalence of common psychiatric disorders in patients with T2D assessed through the Patient Health Questionnaire. Moreover, to evaluate potential risk/protective factors associated with patients’ psychiatric disorders positivity, we performed univariable logistic regression models. Therefore, for each factor, we calculated the Odds Ratio (OR) with 95% Confidence Interval (CI) of being positive to PHQ. Statistical analyses were performed with Stata software, and statistical significance cut-off was set p<0.05.

**The primary objective** will be achieved through the calculation of the prevalence of common psychiatric diseases in patients with T2D.

# References

Istat. Diabetes in Italy, Years 2000-2016. [Internet]. https://www.istat.it/it/files//2017/07/Report_Diabetes_En_def.pdf . [cited 2022, July 09].

American Diabetes Association. 5. Lifestyle Management: Standards of Medical Care in Diabetes-2019. Diabetes Care. 2019 Jan;42(Suppl 1): S46-S60. doi: 10.2337/dc19-S005

Farag YM, Gaballa MR. Diabesity: an overview of a rising epidemic. Nephrol Dial Transplant. 2011 Jan;26(1):28-35. doi: 10.1093/ndt/gfq576

de Groot M, Golden SH, Wagner J. Psychological conditions in adults with diabetes. Am Psychol. 2016 Oct;71(7):552-562. doi: 10.1037/a0040408

Diabetes Canada Clinical Practice Guidelines Expert Committee. Diabetes and Mental Health. Can J Diabetes. 2018 Apr;42 Suppl 1:S130-S141. doi: 10.1016/j.jcjd.2017.10.031

Hussain S, Habib A, Singh A, Akhtar M, Najmi AK. Prevalence of depression among type 2 diabetes mellitus patients in India: A meta-analysis. Psychiatry Res. 2018 Dec;270:264-273. doi: 10.1016/j.psychres.2018.09.037

European Depression in Diabetes (EDID) Research Consortium. Type 2 diabetes mellitus as a risk factor for the onset of depression: a systematic review and meta-analysis. Diabetologia. 2010 Dec;53(12):2480-6. doi: 10.1007/s00125-010-1874-x

Knol MJ, Twisk JW, Beekman AT, Heine RJ, Snoek FJ, Pouwer F. Depression as a risk factor for the onset of type 2 diabetes mellitus. A meta-analysis. Diabetologia. 2006 May;49(5):837-45. doi: 10.1007/s00125-006-0159-x

Roy T, Lloyd CE. Epidemiology of depression and diabetes: a systematic review. J Affect Disord. 2012 Oct;142 Suppl:S8-21. doi: 10.1016/S0165-0327(12)70004-6

Spitzer RL, Kroenke K, Williams JB. Validation and utility of a self-report version of PRIME-MD: the PHQ primary care study. Primary Care Evaluation of Mental Disorders. Patient Health Questionnaire. JAMA. 1999 Nov

10;282(18):1737-44. doi: 10.1001/jama.282.18.1737

American Diabetes Association. Diagnosis and classification of diabetes mellitus. Diabetes Care. 2011 Jan;34 Suppl 1(Suppl 1):S62-9. doi: 10.2337/dc11-S062

**Principal Investigator**

**Prof. Luigi Janiri - Psychiatry Unit Director**

**Area - Neuroscience**


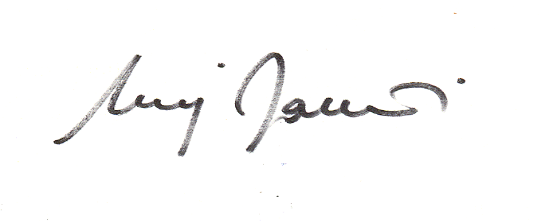
**Fondazione Policlinico Universitario A. Gemelli, Largo A. Gemelli, 8 - 00168 Rome**
