# Supplementary material for: Evaluation of the prevalence of the most common psychiatric disorders in patients with type 2 diabetes mellitus using the patient health questionnaire: results of the cross-sectional “DIA2PSI” study
Source: Acta Diabetol. 2022 Nov 7;60(2):247–55. doi: 10.1007/s00592-022-01993-x (PMC9640892; doi:10.1007/s00592-022-01993-x)
Supplement: Supplementary file 2 — Supplementary file2 (DOCX 16 kb) [file 592_2022_1993_MOESM2_ESM.docx]

**Title**

Evaluation of the prevalence of the most common psychiatric disorders in patients with type 2 diabetes mellitus using the Patient Health Questionnaire: results of the cross-sectional “DIA2PSI” study.

**Journal Name**

Acta Diabetologica

**Author names**

Angelo Emilio Claro ^1,2^*, Clelia Palanza ^3^, Marianna Mazza ^1,2^, Andrea Corsello ^4^, Alessandro Rizzi ^4^, Linda Tartaglione ^4^, Chiara de Waure ^5^, Giuseppe Marano ^1,2^, Simone Piciollo ^6^, Giovanna E. U. Muti Schuenemann ^7^, Marta Rigoni ^8^, Paola Muti ^8^, Alfredo Pontecorvi ^9^, Luigi Janiri ^1,2^, Gabriele Sani ^1,2^, Dario Pitocco ^4^.

*Corresponding Author: email – dott.claro@gmail.com

^1^ Fondazione Policlinico Universitario A. Gemelli IRCCS, Largo Agostino Gemelli, 8 - CAP 00168, Rome, Italy.

^2^ Department of Psychiatry, Università Cattolica del Sacro Cuore, Largo Agostino Gemelli, 8 - CAP 00168, Rome, Italy.

**DIA2PSI STUDY - SOCIODEMOGRAPHIC DATA SHEET**

1 – Given name and last name ……………………………………………………

2 - Date of birth ……………………………………………………

3 - Telephone ……………………………………………………

4 - Email address ……………………………………………………

5 - Gender Male □ Female □

6 - Age ……………………………………………………

7 - Height ……………………………………………………

8 - Current weight ……………………………………………………

9 - School education level? ……………………………………………………

10 - How long have you been diagnosed with type 2 diabetes? ……………………………………………………

11 - How much did you weight when you were diagnosed with type 2 diabetes? ...................................

12 - Are you aware that through lifestyle changes, that is, a significant reduction in body weight, i.e. ≥ 15 kg and by increasing physical activity, it is possible to improve type 2 diabetes (reduce insulin, antidiabetic and antihypertensive requirements), and if you have had T2D for less than 6 years, induce its remission?

                                                                                                                             Yes □ No □

13 - If yes, have you implemented these lifestyle changes?                    Yes □ No □

14 - If yes, which change have you made?

Scheduled exercise □      diet □

body weight reduction □

        other................................................. .................................................. ..................................................

15 - If you answered no to question 13, can you explain why?

your lifestyle is too stressful and you can't follow a diet regularly □

food is an indispensable pleasure for you and you cannot do without it □

you are on a strict diet but still unable to lose weight □

you have too little time to practice on a multi-weekly and sufficient basis □

carry out physical activity several times a week and follow a diet and despite this, you cannot lose weight □

      other reasons…………………………………………………………………………………………………………………………………………

…………………………………………………………………………………………………………………………………………………………..…..

16 - Do you know the importance of mental health over weight control and the prevention of physical illness?                     Yes □ No □

17 - Have you ever been treated by a mental health professional (psychiatrist or psychotherapist)?                     Yes □ No □

18 - Have you ever received psychopharmacological treatments (mood stabilizers, antidepressants,

antipsychotics, sleeping pills, etc.)? Yes □ No □
