# Supplementary material for: Evaluation of the prevalence of the most common psychiatric disorders in patients with type 2 diabetes mellitus using the patient health questionnaire: results of the cross-sectional “DIA2PSI” study
Source: Acta Diabetol. 2022 Nov 7;60(2):247–55. doi: 10.1007/s00592-022-01993-x (PMC9640892; doi:10.1007/s00592-022-01993-x)
Supplement: Supplementary file 3 — Supplementary file3 (DOCX 35 kb) [file 592_2022_1993_MOESM3_ESM.docx]

**Title**

Evaluation of the prevalence of the most common psychiatric disorders in patients with type 2 diabetes mellitus using the Patient Health Questionnaire: results of the cross-sectional “DIA2PSI” study.

**Journal Name**

Acta Diabetologica

**AUTHORS**

Angelo Emilio Claro ^a,b,^*, Clelia Palanza ^c^, Marianna Mazza ^a,b^, Andrea Corsello ^d^, Alessandro Rizzi ^d^, Linda Tartaglione ^d^, Chiara de Waure ^e^, Giuseppe Marano ^a,b^, Simone Piciollo ^f^, Giovanna E. U. Muti Schuenemann ^g^, Marta Rigoni ^h^, Paola Muti ^h^, Alfredo Pontecorvi ^i^, Luigi Janiri ^a,b^, Gabriele Sani ^a,b^, Dario Pitocco ^d^.

*Corresponding Author; email: dott.claro@gmail.com

^a^ Fondazione Policlinico Universitario A. Gemelli IRCCS, Largo Agostino Gemelli, 8 - CAP 00168, Rome, Italy.

^b^ Department of Psychiatry, Università Cattolica del Sacro Cuore, Largo Agostino Gemelli, 8 - CAP 00168, Rome, Italy.

T2D patients from Diabetes Care Unit of the FPUAG

The goal: to evaluate 184 patients diagnosed with T2D according to ADA criteria for diagnosis

300 patients eligible for the study

60 patients refused to partecipate

240 patients recruited

56 patients excluded due to uncertain T2D diagnosis or because PHQ left incomplete

184 patients included

**Fig. 1** Patients’ selection flow chart

**Table 4.** Demographic, and lifestyle predictors of current common psychiatric disorder associated with PHQ positivity determined by univariable logistic regression models.

| **Variables** | **OR (95% CI)** | **p-value** |
| --- | --- | --- |
| **Demographic:** |  |  |
| Sex female versus male | 1.72 (0.94-3.17) | 0.08 |
| Age (for each 1-year increase) | 0.98 (0.95-1.00) | 0.07 |
| **Civil status:** |  |  |
| Civil status (widow-er / single / divorced) vs (cohabiting) | 1.55 (0.79-3.04) | 0.20 |
| ***Residence Location:*** |  |  |
| Urban vs rural location | 0.58 (0.31-1.06) | 0.07 |
| ***Educational level:*** |  |  |
| (High school and degrees) vs (Middle and elementary school) | 0.75 (0.41-1.36) | 0.35 |

**Table 5.** Diabetes mellitus-specific, and psychosocial predictors of current common psychiatric disorder associated with PHQ positivity determined by univariable logistic regression models.

| **Variables** | **OR (95% CI)** | **p-value** |
| --- | --- | --- |
| **Diabetes specific and psychosocial predictors:** |  |  |
| T2D diagnosis (for each one-year increase) | 1.00 (0.99-1.04) | 0.57 |
| BMI, kg/m^2^ (1 point increase) | 1.07 (1.00-1.14) | 0.04 |
| At diagnosis BMI, kg/m^2^ (1 point increase) | 1.05 (1.00-1.10) | **0.06** |
| ***Aware that change lifestyle improves diabetes mellitus?*** |  |  |
| Awareness: yes, vs no | 0.48 (0.13-1.77) | 0.27 |
| Has implemented lifestyle changes: yes, vs no | 0.38 (0.18-0.79) | **<0.01** |
| ***Aware that mental health linked to body health?*** |  |  |
| Aware that mental health linked to body health: yes, vs no | 0.54 (0.28-1.04) | 0.07 |
| ***Previous PSY treatment?*** |  |  |
| yes, vs no | 1.30 (0.67-2.55) | 0.44 |
| ***Previous Pharmaco-PSY treatment?*** |  |  |
| yes, vs no | 2.46 (1.19-5.12) | **0.02** |
| ***Antidiabetic drugs:*** |  |  |
| Antidiabetic Drugs yes vs no | 0.70 (0.34-1.43) | 0.32 |
| Insulin yes vs no | 1.89 (1.02-3.49) | 0.04 |

**Table 6**. Diabetes mellitus-specific predictors of current common psychiatric disorder associated with PHQ positivity determined by univariable logistic regression models.

| **Variables** | **OR (95% CI)** | **p-value** |
| --- | --- | --- |
| ***T2D related complications:*** |  |  |
| yes, vs no | 1.53 (0.82-2.83) | 0.18 |
| ***Number of T2D related complications:*** |  |  |
| Number of T2D related complications (1 point increase) | 1.17 (0.88-1.55) | 0.28 |
| T2D related complications 1 vs 0 | 1.39 (0.68-2.83) | 0.36 |
| T2D related complications 2 vs 0 | 1.94 (0.86-4.37) | 0.11 |
| T2D related complications 3 vs 0 | 1.36 (0.45-4.12) | 0.58 |
| T2D related complications 4 vs 0 | 0.88 (0.07-10.16) | 0.91 |
| ***HbA1C:*** |  |  |
| HbA1c (1 point increase) | 1.07 (0.89-1.28) | 0.47 |
| % HbA1C ≥ 6.5% versus < 6.5% | 0.95 (0.51-1.75) | 0.87 |
| ***Hypertension:*** |  |  |
| Hypertension yes vs no | 1.63 (0.80-3.30) | 0.18 |
